# Two Parallel Olfactory Pathways for Processing General Odors in a Cockroach

**DOI:** 10.3389/fncir.2017.00032

**Published:** 2017-05-05

**Authors:** Hidehiro Watanabe, Hiroshi Nishino, Makoto Mizunami, Fumio Yokohari

**Affiliations:** ^1^Division of Biology, Department of Earth System Science, Fukuoka UniversityFukuoka, Japan; ^2^Research Institute for Electronic Science, Hokkaido UniversitySapporo, Japan; ^3^Faculty of Science, Hokkaido UniversitySapporo, Japan

**Keywords:** insect, olfaction, parallel processing, projection neurons, temporal pattern, intracellular recording, simultaneous intracellular recording, antennal lobe

## Abstract

In animals, sensory processing via parallel pathways, including the olfactory system, is a common design. However, the mechanisms that parallel pathways use to encode highly complex and dynamic odor signals remain unclear. In the current study, we examined the anatomical and physiological features of parallel olfactory pathways in an evolutionally basal insect, the cockroach *Periplaneta americana*. In this insect, the entire system for processing general odors, from olfactory sensory neurons to higher brain centers, is anatomically segregated into two parallel pathways. Two separate populations of secondary olfactory neurons, type1 and type2 projection neurons (PNs), with dendrites in distinct glomerular groups relay olfactory signals to segregated areas of higher brain centers. We conducted intracellular recordings, revealing olfactory properties and temporal patterns of both types of PNs. Generally, type1 PNs exhibit higher odor-specificities to nine tested odorants than type2 PNs. Cluster analyses revealed that odor-evoked responses were temporally complex and varied in type1 PNs, while type2 PNs exhibited phasic on-responses with either early or late latencies to an effective odor. The late responses are 30–40 ms later than the early responses. Simultaneous intracellular recordings from two different PNs revealed that a given odor activated both types of PNs with different temporal patterns, and latencies of early and late responses in type2 PNs might be precisely controlled. Our results suggest that the cockroach is equipped with two anatomically and physiologically segregated parallel olfactory pathways, which might employ different neural strategies to encode odor information.

## Introduction

Information processing along parallel pathways is a common feature of animal sensory systems, enabling fast and reliable representations of highly complex and dynamic sensory information. Parallel sensory pathways typically extract and process different types of stimulus parameters. In the vertebrate olfactory system, general and pheromonal odors are processed in the main olfactory and vomeronasal pathways, respectively (Tirindelli et al., 2009). Within the main olfactory pathway, mitral and tufted cells in the olfactory bulb process odor qualities and timing information, respectively (Nagayama et al., 2004; Igarashi et al., 2012).

Because the olfactory bulb of vertebrates and the antennal lobe (AL) of insects are functionally similar neural substrates, the insect olfactory system consisting of a small number of identifiable neurons provides a useful model for studying the neural basis of parallel olfactory processing (Hildebrand and Shepherd, 1997; Knaden and Hansson, 2014). In insects, olfactory sensory neurons (OSNs) are housed in the antennal sensilla and project to glomeruli in the AL. In each glomerulus, a large number of OSNs, which express a cognate type of odorant receptor relay onto a moderate number of secondary olfactory interneurons, projection neurons (PNs), and local interneurons (LNs). PNs send olfactory information to higher brain centers, mushroom bodies (MBs) and lateral horns (LHs). Conversely, LNs in the AL interconnect glomeruli and contribute to odor-induced spatio-temporal activity patterns of PNs (Wilson et al., 2004; Watanabe et al., 2012a).

Two categories of parallel olfactory pathways have been proposed in insects (Galizia and Rössler, 2010): “segregated parallel pathways,” which process different odors in different pathways, and “dual parallel pathways,” which process different parameters of a given odor. For example, the pathway for processing species-specific odors, such as pheromones, is anatomically segregated from the pathway for processing general odors from antennal sensilla to higher brain centers (Burrows et al., 1982; Nishikawa et al., 2012; Nishino et al., 2012a). The dual parallel pathways have been observed in hymenopteran insects. In honeybees and ants, the axons of PNs innervating glomeruli in the dorsal AL hemilobe run through the lateral AL tract (l-ALT), whereas those of PN axons innervating glomeruli in the ventral AL hemilobe run through the medial ALT (m-ALT) (Kirschner et al., 2006; Zube et al., 2008). Because both l-ALT and m-ALT PNs receive olfactory inputs in part from common sensilla (Kelber et al., 2006) and their projection fields partially overlap, their anatomical subdivisions are unclear. A given odor stimulus recruits both PN types, and studies have suggested that l-ALT and m-ALT PNs might extract different parameters of a given olfactory stimulus (Muller et al., 2002; Krofczik et al., 2008; Galizia and Rössler, 2010; Rössler and Zube, 2011; Brill et al., 2013, 2015; Rössler and Brill, 2013). However, the mechanisms by which parallel pathways extract and encode different parameters of olfactory information remain unclear.

The current study sought to determine the neural mechanisms underlying parallel olfactory processing in insects. In the cockroach *Periplaneta americana*, the AL equips two sex pheromone-sensitive glomeruli and 203 ordinary glomeruli which process general odors. The ordinary glomeruli have been classified into two glomerular groups: the antero-dorsal and postero-ventral groups (Watanabe et al., 2010). Because the antero-dorsal and postero-ventral group glomeruli receive olfactory inputs from OSNs in different types of sensilla that exhibit different odor spectra, they are morpho-functionally segregated (Fujimura et al., 1991; Watanabe et al., 2012a,b). The current study revealed that olfactory signals processed in postero-ventral and antero-dorsal group glomeruli are relayed by two morphologically distinct types of uniglomerular PNs, termed type1 and type2 PNs, respectively (Strausfeld and Li, 1999a; Watanabe et al., 2012a). We used two approaches that provided insight into the neural mechanisms underlying parallel olfactory processing: (1) we examined the anatomical and physiological features of PNs in each pathway and (2) we performed simultaneous intracellular recording from two different types of PNs.

## Materials and Methods

### Experimental Animals

Male adult cockroaches *P. americana* were obtained from laboratory colonies maintained under a 12:12-h light:dark cycle at 28°C in Fukuoka University.

### Tracer Application to Projection Neurons and OSN Afferents

After cockroaches were anesthetized on ice, the head capsule was incised and fixed to a wax plate anterior side up. The cuticle between the two antennae was squarely cut and the overlaying muscles and tracheae on the brain were removed. In the cockroach, axons of all uniglomerular PNs run through the m-ALT or nearby tracts (Malun et al., 1993; Strausfeld and Li, 1999a). For retrograde staining of PNs, a tapered glass electrode coated with moisture-absorbed crystals from micro-emerald (dextran fluorescein with biotin, 3000 MW, D-7156, ThermoFisher Scientific, Waltham, MA, USA) was manually inserted into the m-ALT. After application of the fluorescent dye, the opening of the head was covered with a previously cut square of cuticle. Antennal afferents then underwent anterograde staining to visualize glomeruli as previously described (Nishino et al., 2009; Watanabe et al., 2010). The antennal nerve on the ipsilateral side was exposed and excised at the flagellar base. The proximal cut end of the antennal nerve was inserted into a tapered glass capillary filled with a 10% aqueous solution of micro-ruby (dextran tetramethyl rhodamine with biotin, 3000 MW, D-7162, ThermoFisher Scientific). The specimen was incubated in a moist chamber under dark conditions at 4°C for 2 days. After incubation, the brain was dissected from the head capsule. The isolated brain was fixed in a 4% formaldehyde solution at 4°C for 3–5 h, dehydrated in an ascending ethanol series (from 70% to 100%), then cleared in methyl salicylate.

### Single and Simultaneous Intracellular Recordings

The method used for intracellular recording and staining from individual PNs of the cockroach was modified from methods reported in our previous studies (Nishino et al., 2003, 2011, 2012a; Watanabe et al., 2012a). Cockroaches were briefly anesthetized and mounted on experimental chambers with low-melting point wax. Each antenna was immobilized by threading a plastic ring (diameter: 1 mm). The cuticle between the two antennae was opened, and the brain was exposed. After the brain sheath had been softened with Actinase E (Kaken Seiyaku, Tokyo, Japan), the brain and platinum ground electrode were immersed in a cockroach saline solution (NaCl 210.2 mM, KCl 3.1 mM, CaCl_2_ 1.8 mM, NaH_2_PO_4_ 0.2 mM, Na_2_HPO_4_ 1.8mM, pH 7.2). To stabilize the brain, a glass rod was inserted into the cavity formed by removal of the esophagus.

**FIGURE 1:**
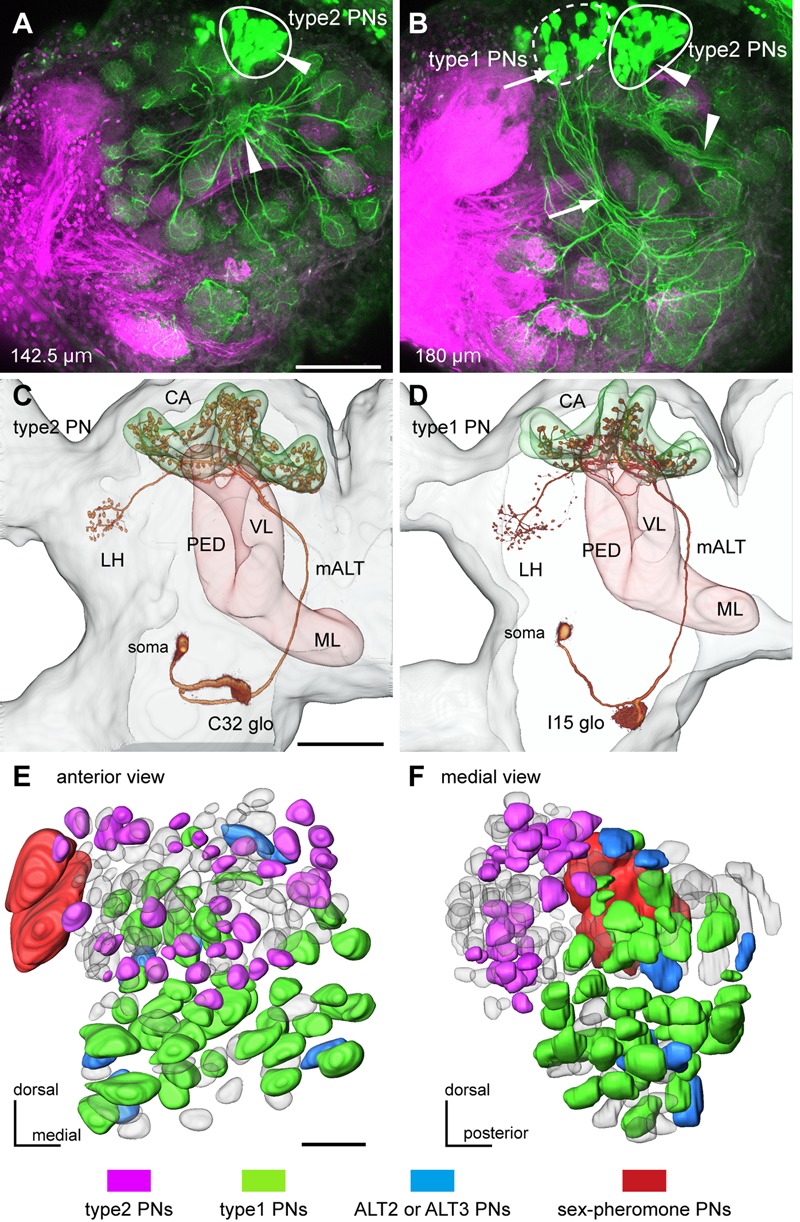
**Two major types of uniglomerular projection neurons (PNs) in the cockroach brain. (A,B)** Mass staining of PNs in the antennal lobe (AL). PNs (green) were stained by dye injection into the medial ALT (m-ALT) and olfactory sensory neurons (OSNs) (magenta) were labeled by anterograde staining from the antennal nerves. Axons of PNs form two different bundles in the AL (arrowheads and arrows in **A,B**), and their cell bodies separately cluster in the dorsal region of the AL. Loci of clusters of cell bodies correspond to the two major types of PNs; type1 and type2 PNs. Depth of serial optical image from anterior surface is indicated in each panel. **(C,D)** 3D images of two major types of uniglomerular PNs. Axons of both type2 PN **(C)** and type1 PN **(D)** run through the m-ALT, anteriorly to the mushroom bodies (MB) peduncle (PED) and terminate in both MB calyces (CA) and lateral horn (LH). **(E,F)** PN type-specific glomerular organization, viewed anteriorly **(E)** and medially **(F)**. Glomeruli innervated by intracellularly stained PNs are colored according to PN types. Type2 PNs specifically arborize in the antero-dorsal group glomeruli (magenta in **E,F**), whereas type1 PNs arborize in the postero-ventral group glomeruli (green in **E,F**). Glomeruli innervated by ALT2 and ALT3 uniglomerular PNs, which have been identified by Malun et al. (1993), are colored blue. Two macroglomeruli innervated by sex pheromone-sensitive PNs are colored red. Bars in **(A,E)** = 100 μm, bar in **(C)** = 200 μm.

A borosilicate glass microelectrode pulled by a laser puller (P-2000; Sutter Instruments, Novato, CA, USA) was filled with 8% Lucifer Yellow (Sigma, St. Louis, MO, USA) or 10 mM Alexa 647 (ThermoFisher Scientific) in 1 M LiCl (aqueous). An electrode was inserted into the cluster of PN somata located in the dorsal region of the AL (**Figures 1A,B**; Distler and Boeckh, 1997a,b; Watanabe et al., 2012a). In simultaneous intracellular recordings from two different PNs, two electrodes were filled with different fluorescent dyes (Lucifer Yellow and Alexa 647) and separately inserted into the ipsilateral AL. The neural activity of individual neurons was amplified (MEZ8301, Nihon Kohden, Tokyo, Japan) and displayed on an oscilloscope. Spikes were digitized by a PowerLab data acquisition system (AD Instruments Japan Inc., Nagoya, Japan).

### Olfactory Stimulation

To attain PN response data efficiently, we carefully chose nine different odors that collectively cover the broad spectra of sensory neuronal responses. In the cockroach *P. americana*, most OSNs responding to general odors have been classified into eight groups based on similarities in the response spectra (Fujimura et al., 1991). In the current study, nine mono-molecule odorants, each eliciting strong excitatory effects in one of the eight OSN groups, were selected: pentanol, hexanol, octanol, nonanol, phenyl acetate, cineol, santalol, terpineol, and heptanoic acid. Pure solutions of each odorant were diluted 10 times with paraffin oil, and a small piece of filter paper was soaked with 40 μL of one of the solutions and inserted into each nozzle.

Fresh air taken from outdoors via a diaphragm pump was cleaned with a cotton and charcoal filter and divided into two tubes; one passed through three bottles filled with distilled water (humidified air) and the other passed through three bottles filled with silica-gel (dried air). We used air at 50% relative humidity by mixing the humidified and dried air. The flow-rate of air within the tube was maintained at 1 L/min using a flowmeter. The tube was further divided into nine connected to nine glass nozzles containing nine different odorants. Odorant nozzles and a blank (control) nozzle were arbitrarily selected for stimulation by manual valve operation. The nozzle tip (5 mm diameter) was positioned approximately 10 mm from approximately the 30th antennal flagellomere, and the air around the preparation was continuously exhausted by a duct located in front of the antenna tip. The three-way solenoid valve was controlled by a stimulator (SEN7203, Nihon Kohden, Tokyo, Japan). We defined the timing of the solenoid valve opening by a stimulator as the odor onset. The stimulus period was set at 1 s. We designed experiment to minimalize the number of olfactory stimulations to avoid the deterioration of recorded PN: each odorant was presented twice with a 30-s interval. After stimulation with a given odorant, a new nozzle containing another odorant was moved to the same position as the previous nozzle.

### Confocal Observations and Three-Dimensional Reconstruction

After recording olfactory responses, the neuron was filled with fluorescent dye by injecting a hyperpolarizing current. After intracellular staining, the electrode was removed from the brain, and the head capsule was fixed on a wax plate. Antennal afferents then underwent anterograde staining using micro-ruby, as previously described. Contact between the specimen and the dye was maintained in a humid chamber at 4°C overnight. Subsequently, the double- or triple-stained brain was dissected from the head capsule. The isolated brain was fixed, dehydrated and cleared, as previously described.

The cleared specimens were examined with a confocal laser scanning microscope (LSM-510; Carl Zeiss, Jena, Germany) equipped with Argon and Helium-Neon lasers. Single neurons labeled by intracellular injection of Lucifer Yellow and/or Alexa 645 were, respectively, visualized using an Argon laser with a band-pass emission filter (505–550 nm) and/or a Helium-Neon laser with a long-pass emission filter (>650 nm). In addition, sensory afferents labeled with micro-ruby were acquired using a Helium-Neon laser with a band-pass emission filter (560–615 nm). Images were obtained using three different objectives: a Plan-Apochromat 10×/0.7 and 20×/0.8 objectives for low magnification images, and an oil-immersion Plan-Neofluar 40×/1.3 were used for capturing high magnification images. All images were captured as 1024 × 1024 pixels. A series of TIFF-formatted optical sections were processed using image processing software (Amira 6.0, TGC, Berlin, Germany). Labeled neurons were traced with an image segmentation tool in Amira. The contrast and brightness of all images were adjusted appropriately using Adobe Photoshop CS3 and Illustrator CS3.

### Terminology

In the male cockroach AL, all 203 ordinary glomeruli and two macroglomeruli can be unambiguously identified by the innervation patterns of T1 to T10 sensory tracts (Watanabe et al., 2010). The nomenclatures and detailed morphological features of glomeruli and glomerular groups were described in our previous studies (Watanabe et al., 2010, 2012b). Specifically, ordinary glomeruli belonging to the T1-T4 groups were morphologically, functionally, and developmentally segregated from those belonging to the T5-T10 groups (Salecker and Boeckh, 1995; Watanabe et al., 2010, 2012b). We termed the former and latter groups the ‘antero-dorsal’ and ‘postero-ventral glomerular groups,’ respectively. The PN types were named according to terminology used in previous anatomical studies (Malun et al., 1993; Strausfeld and Li, 1999a; Nishino et al., 2012a). Each PN was named based on the innervating glomerulus. The medial and lateral calyces of the MB were defined as input sites of Kenyon cells (KCs), and each calyx was divided into four zones (I–III, IIIA) from the periphery to the base (Mizunami et al., 1998; Strausfeld and Li, 1999a,b). Brain orientation is shown with reference to the body axis. Nomenclatures and abbreviations of brain structures followed those established in the systematic nomenclature for the insect brain (Ito et al., 2014).

### Data Analyses

To quantify spike numbers during a given time period, we used functions attached in Spike 2 ver.8.08 (CED, Cambridge, England). Odor responses were measured as an increase in spike frequency from the spontaneous level; R-R_0_, where R or R_0_ was the number of spikes during the 1-s odor stimulation or during the 1-s pre-stimulation period, respectively. In each PN, odor intensity to a given odor was represented as the average R-R_0_ of two trials. To identify odor response properties of recorded PNs, we performed hierarchical cluster analysis using the free programming software R v.3.3.2 (R Foundation for Statistical Computing, Vienna, Austria). Based on “R-R_0_” to nine tested odors, recorded PNs were classified into several groups using the cluster dendrogram expressed by Ward’s method. Odor responses were regarded as excitatory if the R was more than two times higher than R_0_ in the two trials. In each recording, we quantified the number of effective odors that elicited excitatory responses to the PN. The recruitment rates of PNs were shown as a percentage of odor-activated PNs per odor. The percentages were separately calculated in type1 and type2 PNs. The number of effective odors and recruitment rates were statistically compared using the chi-square test or paired *t*-test.

Temporal activity patterns elicited by a given odor stimulus were summarized as raster plots and accumulated histograms with a bin of 10 ms. After normalizing the histograms by dividing the number of spikes of each bin by the number of samples, we statistically compared histograms using the Kolmogorov–Smirnov test (KS-test). To compare differences of odor-induced temporal activity patterns across PN types and across odors, we performed the hierarchical cluster analysis (Ward’s method) and the principal component analysis (PCA) using the “cluster” package in R software. In these analyses, we used excitatory responses to four different odorants (hexanol, octanol, phenyl acetate, and cineol) obtained by single intracellular recordings. In each response, the spike array during the 1-s odor stimulation was represented as a peri-stimulus time histogram (PSTH) with a bin of 20 ms. We classified odor-induced PSTHs into several clusters based on the cluster dendrogram. Using the “clusplot” package in R software, we plotted scores of the first two principal components (PCs) obtained from PCA. Distributions of the first two PCs are shown as box plots and variances of PCs were statistically compared using the *F*-test or two-way ANOVA test.

Cluster analysis using PSTHs strongly revealed two different odor-induced on-phasic responses in type2 PNs. To evaluate odor-induced on-phasic responses in type2 PNs in greater detail, we used 160 responses from 16 different type2 PNs that exhibited excitatory responses to more than five different odors. Olfactory responses are shown as time-courses of instantaneous spike frequencies. The instantaneous spike frequency was defined as reciprocals of time intervals between successive spikes. In each olfactory response, we identified a spike that a time-course of instantaneous spike frequencies peaked or plateaued, which was termed a “peak spike”. Because the odor arrival time to antenna varied (10–20 ms) across specimens, the timing of each peak spike was corrected as follows; “t_peak_ – t_first_”, where t_peak_ was a time of the peak spike from the odor onset (timings of opening of solenoid valves) and t_first_ was a time of earliest odor-induced spike in the specimen. Peak spikes were classified into three groups based on its instantaneous spike frequencies: >300 Hz, 200–300 Hz, and <200 Hz. One-way ANOVA and *post hoc* Tukey test was used to compare time distribution patterns of peak spikes in the three groups.

Using the standard glomeruli map in the male cockroach AL (Watanabe et al., 2010, 2012b), we colored glomeruli based on anatomical and physiological properties of innervating PNs.

## Results

### Segregated Parallel Pathways from the Periphery to the Higher Brain Centers

In the cockroach, axons of uniglomerular PNs run through the m-ALT and nearby tracts (ALT-2 and ALT-3), and terminate in the medial and lateral calyces (CA) of the MB first and then in the LH (Malun et al., 1993; Strausfeld and Li, 1999a; Nishino et al., 2012a). Retrograde staining of PNs revealed all glomeruli were innervated by PNs running m-ALT and nearby tracts in the cockroach (**Figures 1A,B**). Except for two sex pheromone-receptive macroglomeruli that were each innervated by multiple PNs (Nishino et al., 2011), individual ordinary glomeruli were in principle innervated by a single uniglomerular PN (**Figures 1A,B**; Ernst and Boeckh, 1983). PNs innervating the antero-dorsal group glomeruli (arrowheads in **Figures 1A,B**), and those innervating the postero-ventral group glomeruli (arrows in **Figure 1B**) formed two distinct axon bundles in the AL.

Based on intracellular staining and Golgi staining, several morphological types of PNs have been identified in the cockroach brain (Malun et al., 1993; Strausfeld and Li, 1999a). We identified type1 and type2 PNs based on their terminal zones in the calyces; terminal blebs of each type 1 PN are broadly distributed within zones III and IIIA of calyces, and those of each type2 PN are concentrated to the zone I (Strausfeld and Li, 1999a; Takahashi et al., 2017). In the current study, we intracellularly stained 115 type1 PNs, 63 type2 PNs, and 11 PNs of other types. Type1 and type2 PNs have cell bodies in the antero-dorsal region of the AL (**Figures 1A–D**). The results revealed that axons of type2 and type1 PNs ran through separate bundles in the AL (**Figures 1A–D**). After running through the m-ALT, axons of type1 and type2 PNs turned laterally in the anterior side of the MB pedunculus, and finally terminated in the MB calyces and the LH (**Figures 1C,D**). Based on intracellular staining results, we mapped 106 glomeruli with respect to innervating PN types (**Figures 1E,F**). Type2 and type1 PNs had dendrites in the antero-dorsal and postero-ventral group glomeruli, respectively (**Figures 1E,F**). Among the 106 glomeruli analyzed, only 10 postero-ventral group glomeruli were connected with the ALT-2 or ALT-3 PNs previously identified (Malun et al., 1993), and two macroglomeruli were connected with sex pheromone-sensitive PNs that terminated in specific regions of higher brain centers (Nishino et al., 2011, 2012a). Here, we focused on the two major streams formed by type1 and type2 PNs.

**FIGURE 2:**
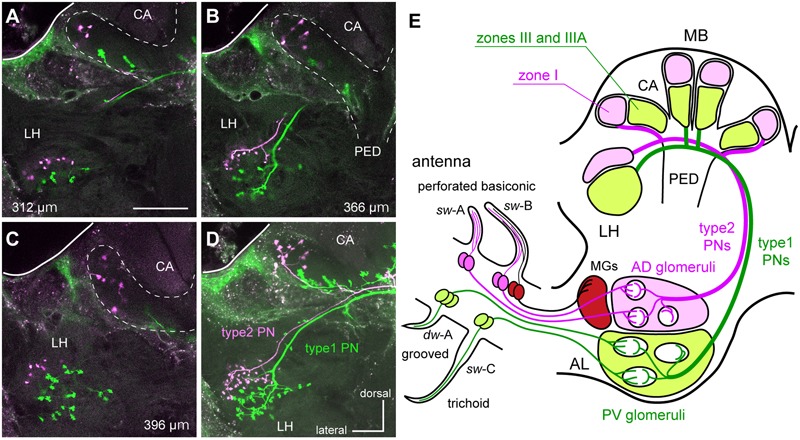
**The segregated parallel pathways from the periphery to higher brain centers in the cockroach brain. (A–D)** A type1 PN (green) and a type2 PN (magenta) differentially labeled in the protocerebrum. Serial optical sections **(A–C)** and a stacked image **(D)** reveal that terminal regions of type1 PN are segregated from those of the type2 PN in the MB calyces (CA) and the LH. Depths of the serial optical images from anterior to posterior are indicated in each of panels **(A–C)**. Twenty-five serial optical images obtained every 4-μm are stacked in **(D)**. Bar in **(A)** = 100 μm. **(E)** Schematic drawing of parallel pathways from the periphery to higher brain center in the cockroach brain. OSNs in perforated basiconic sensilla (*single-walled* A [*sw*-A] and *sw*-B) selectively terminate in antero-dorsal group glomeruli (AD glomeruli), whereas those in trichoid sensilla (*sw-*C) and grooved basiconic sensilla (*double-walled* A [*dw*-A]) projects to the postero-ventral group glomeruli (PV glomeruli: Watanabe et al., 2012b). Therefore, type1 PNs with dendrites in PV glomeruli and type2 PNs with dendrites in AD glomeruli form two segregated parallel pathways from the periphery to higher brain centers. Nomenclatures of MB calyces were described in previous articles (Mizunami et al., 1998; Strausfeld and Li, 1999a,b). MGs, macroglomeruli; PED, pedunculus.

Dual intracellular staining unambiguously revealed that axon terminals of type1 PNs were spatially segregated from those of type2 PNs not only in the MB calyces, but also in the LH (**Figures 2A–D**). Type1 PNs terminated in zones III and IIIA of the MB calyces and the central region of the LH (**Figures 2A–D**). Conversely, type2 PNs terminated in the peripheral-most region of the calyces (zone I) and the antero-dorso-lateral region of the LH (**Figures 2A–D**). Segregation of axon terminals between type1 and type2 PNs was observed in all dual intracellular staining specimens (*n* = 5). In previous cockroach studies, OSNs in antennal perforated basiconic sensilla were reported to terminate specifically in the antero-dorsal group glomeruli, whereas those in the trichoid and grooved basiconic sensilla terminated in postero-ventral group glomeruli (**Figure 2E**; Watanabe et al., 2012b). Thus, type1 and type2 PNs appear to receive sensory inputs from different types of antennal sensilla (**Figure 2E**). The current results suggested that general odors are processed by distinct parallel olfactory pathways from the periphery to the higher brain centers in the cockroach brain (**Figure 2E**).

### Odor-Specificities of Two Different Types of PNs

**FIGURE 3:**
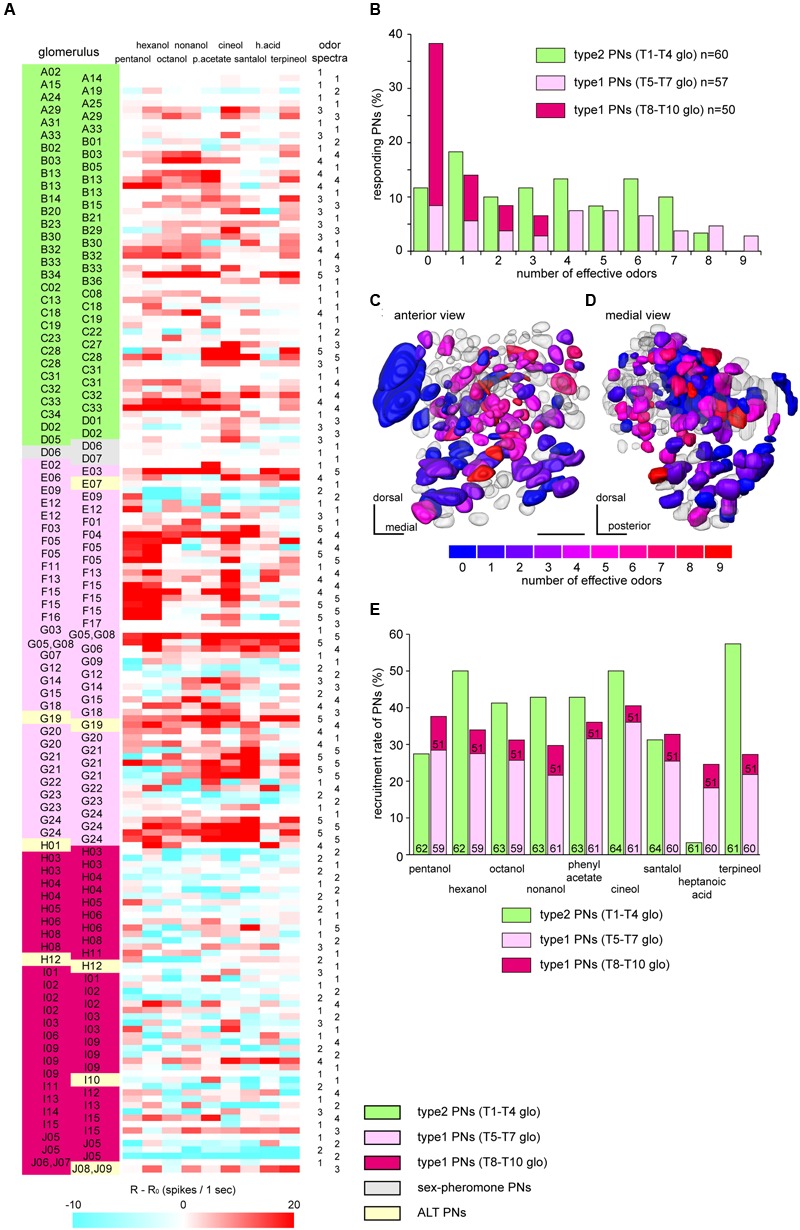
**Classification of PNs according to odor-specificities. (A)** Response intensities to nine tested odorants in recorded PNs. We summarized response intensities to nine odorants, PN types, innervating glomeruli and odor spectra groups of 178 identified PNs. Response intensities are colored according to the values of “R-R_0_” values (see “Materials and Methods”). PNs are classified into five odor spectra groups by cluster analyses using Ward’s method (**Supplementary Figure S1**). Post-recording visualization revealed innervating glomeruli and PN types. Innervating glomeruli are identified based on the 3D-map of the cockroach AL (Watanabe et al., 2010). **(B)** Odor-specificities of PNs. A zero effective odor number means that PNs did not show any excitatory responses to all tested nine odors. The percentages of responding PNs were calculated in each of pathways (type2 PNs, green bars, *n* = 60; type1 PNs, red bars, *n* = 107). Within type1 PNs, PNs arborizing in the T8–T10 group glomeruli (dark red bars, *n* = 50) show higher odor-specificity than those arborizing in the T5–T7 group glomeruli (light red bars, *n* = 57). **(C,D)** Glomerular maps of odor-specificities, viewed anteriorly **(C)** and medially **(D)**. Glomeruli innervated by recorded PNs are colored according to the number of effective odors. The colder color represents higher odor-specificity. When PNs innervated the same glomerulus and exhibited different effective odor numbers, we colored the glomerulus based on the largest effective odor number. **(E)** Recruitment rates of PNs. The percentages of responding type2 PNs (green bars) and type1 PNs (red bars) per odor are shown as recruitment rates. Numbers denoted in bars are sample numbers used in the analysis.

We successfully recorded intracellular responses to nine odorants in 184 PNs (including those innervating the same glomerulus). Among them, we unambiguously identified glomeruli with dendrites of 60 type2 PNs, 107 type1 PNs, and 11 other types of PNs (**Figure 3A**). In each recording, we calculated response intensities to nine odorants by subtracting the number of spikes during the 1-s pre-stimulation period (R_0_) from the number of spikes during the 1 s odor stimulation (R). Endogenous spike activity of each type2 PN was nearly silent (average: 1.94 Hz; 60 type2 PNs; R_0_, *n* = 1077), whereas type1 PNs exhibited spontaneous spike activities of various frequencies (average: 6.30 Hz; 107 type1 PNs; R_0_, *n* = 1926). Therefore, type1 PNs exhibited not only excitatory, but also inhibitory responses to odors (blue colors in **Figure 3A**). We performed cluster analysis using response intensities to nine odorants, and classified recorded PNs into five different odor spectra groups (**Figure 3A** and **Supplementary Figure S1**). The “one glomerulus – one PN” relationship is applicable to the cockroach (Boeckh and Ernst, 1987; **Figures 1A,B**), and PNs originating from the same glomerulus tended to belong to the same odor spectra group (**Figure 3A**). Therefore, response properties of PNs were conserved among individuals. Among five odor spectra groups, group 2, which exhibited inhibitory responses to nine tested odors, and group 5, which exhibited strong excitatory responses to many odors, were predominantly composed of type1 PNs. Thus, type1 and type2 PNs tend to belong to different odor spectra groups (χ^2^-test, *P* = 9.95 × 10^-8^, *df* = 4, χ^2^ = 38.25).

Odor-specificities of both PN types were estimated by the number of effective odors (**Figures 3B–D**). The result revealed that 48% of recorded type2 PNs (*n* = 60) exhibited excitatory responses to more than four different odors out of nine tested odors. Conversely, 38% of recorded type1 PNs (*n* = 107) exhibited no responses to nine tested odors. On average, a single type2 PN was activated by approximately 3.39 of the nine test odors (median, 3), whereas a single type1 PN on average responded to approximately 2.48 of the test odors (median, 1). These results suggested that type2 PNs were significantly broader tuned than type1 PNs to the nine tested odors (χ^2^-test, *P* = 2.26 × 10^-3^, *df* = 9, χ^2^= 25.29). Additionally, type1 PNs exhibited different odor-specificities depending on innervating glomerular groups (**Figures 3B–D**). On average, 61% of recorded type1 PNs innervating the T5–T7 group glomeruli exhibited excitatory responses to more than four different odorants (*n* = 57; average, 4.07; median, 4), whereas 64% of recorded type1 PNs innervating the T8–T9 group glomeruli exhibited no responses to nine tested odors (*n* = 50; average, 0.63; median, 0). These results suggested that odor-specificities of type1 PNs innervating the T8–T10 group glomeruli were significantly higher than type1 PNs innervating the T5–T7 group glomeruli (χ^2^-test, *P* = 5.99 × 10^-7^, *df* = 9, χ^2^= 46.01).

Next, we quantified all PNs activated by each tested odor and calculated the recruitment rates of type1 and type2 PNs to a given odor stimulus (**Figure 3E**). Generally, a given odor recruited both type1 and type2 PNs. The average proportion of activated type2 PNs (38.47%) was not significantly different from type1 PNs (32.86%) (paired *t*-test, *P* = 0.278, *df* = 8, *T* = 1.16). However, from the view-point of each odor, the recruitment rates between type1 and type2 PNs varied (χ^2^-test, *P* = 3.31 × 10^-5^, *df* = 8, χ^2^= 34.49). For example, heptanoic acid specifically activated type1 PNs, and there were only two type2 PNs that exhibited excitatory responses to the odor. The result matched the spatial activation patterns of glomeruli observed in the previous imaging study (Watanabe et al., 2012a). Recruitment rates of type1 PNs innervating the T5–T7 group glomeruli (average, 26.25%) were significantly higher than those of type1 PNs innervating the T8–T10 group glomeruli (average, 6.37%) (paired *t*-test, *P* = 1.15 × 10^-5^, *df* = 8, *T* = 8.59). Moreover, type1 PNs exhibited different response profiles depending on the glomerular groups.

### Temporal Activity Patterns of Two Different Types of PNs

**FIGURE 4:**
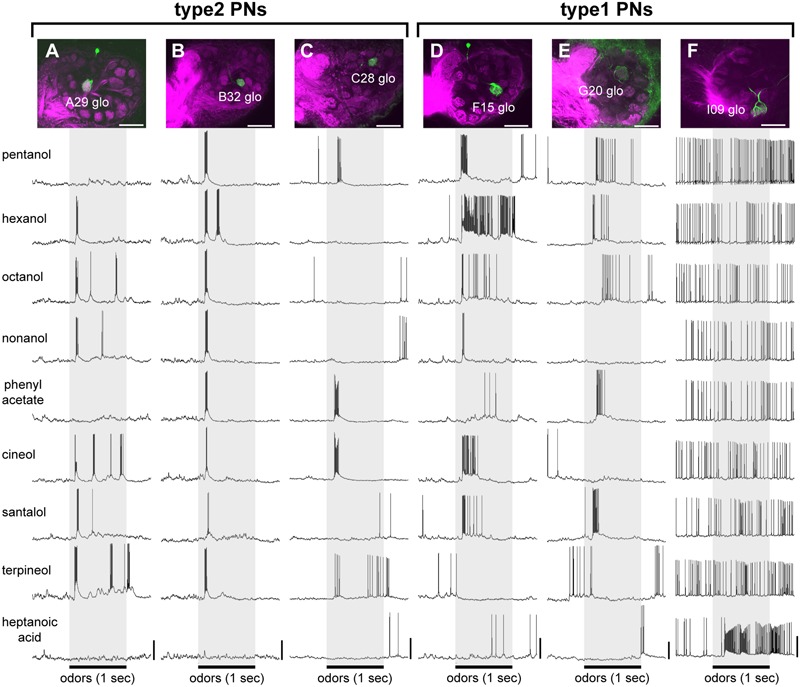
**Typical olfactory responses of two different types of PNs. (A–F)** Olfactory responses of three type2 PNs **(A–C)** and three type1 PNs **(D–F)**. Each of the recorded PNs (green) has its dendrites in a single glomerulus (magenta) belonging to different glomerular groups [laser scanning microscope (LSM) images in **A–F**]. During intracellular recording, nine odors were presented to the antenna. The 1-s olfactory stimuli are indicated by gray boxes. White bars in LSM images = 100 μm; vertical bars = 20 mV.

We show olfactory responses of three type2 PNs and three type1 PNs belonging to different glomerular groups in **Figure 4**. Generally, type2 PNs exhibited phasic on-responses and subsequent cessations of spike activity to effective odors (**Figures 4A–C**). The excitatory on-response persisted for approximately 50–100 ms. In some specimens, the excitatory phase and the break phase alternately appeared during the stimulus period (**Figures 4A,B**). In each type2 PN, different olfactory stimuli induced phasic on-responses. The excitatory on-responses were commonly observed among type2 PNs, though effective odors differed among different PNs (**Figures 3A, 4A–C**). In contrast to type2 PNs, different olfactory stimuli induced different temporal activity patterns in each of the type1 PNs (**Figures 4D–F**). For example, type1 PNs innervating the F15 glomerulus exhibited phasic on-response to nonanol but tonic response to hexanol (**Figure 4D**). Olfactory responses of type1 PNs tended to be more temporally complicated compared with the phasic on-responses of type2 PNs. In addition to excitatory responses, inhibitory responses characterized with prominent IPSPs were observed in type1 PNs that exhibited spontaneous spike activities (**Figures 3A, 4F**).

**FIGURE 5:**
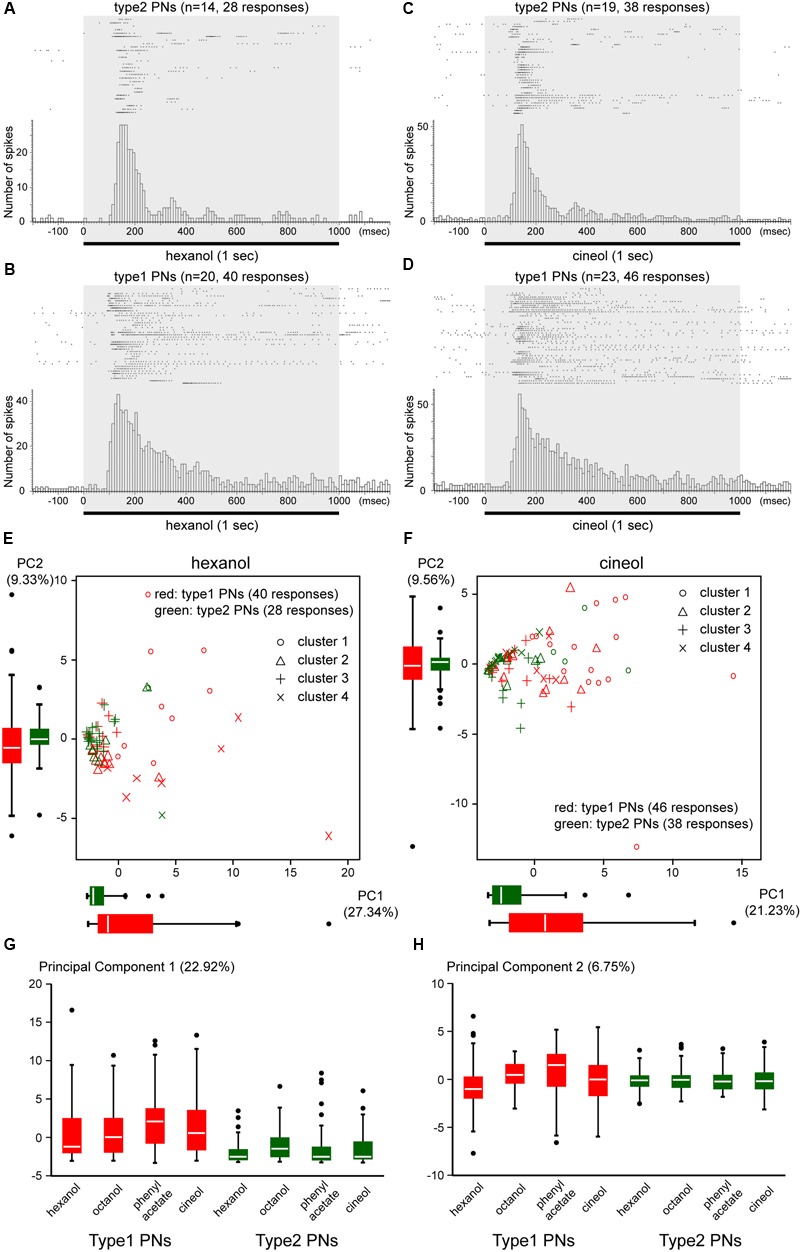
**Temporal activity patterns of type1 and type2 PNs. (A–D)** Temporal activity patterns of type1 and type2 PNs elicited by a given odor. Responses of type2 PNs and type1 PNs to hexanol (**A**, 28 responses from 14 type2 PNs; **B**, 40 responses from 20 type1 PNs) and cineol (**C**, 38 responses from 19 type2 PNs; **D**, 46 responses from 23 type1 PNs) are shown as raster plots (top) and cumulative histograms with a bin of 10 ms (bottom). All PNs were stimulated with a 1-s pulse of odor stimulus (gray box). Raster plots show that both hexanol and cineol evoke on-phasic responses in type2 PNs and various response patterns in type1 PNs. **(E,F)** Differences in temporal activity patterns between type1 and type2 PNs. Based on the peri-stimulus time histograms (PSTHs) with a bin of 20 ms, 68 responses to hexanol **(E)** and 84 responses to cineol **(F)** are, respectively, clustered into four clusters (**Supplementary Figures S2, S3**). Responses of type1 and type2 PNs are, respectively, plotted red and green using the first two PCs (PC 1 and PC 2). Distributions of PC 1 and PC 2 in type1 and type2 PNs are shown as box plots. Each marker represents the response clusters (**Supplementary Figures S2, S3**). PC 1 and PC 2 explain 36.67% of the point variability in **(E)** and 30.79% in **(F)**. **(G,H)** Differences of temporal activity patterns across odors. Based on PSTHs of 284 responses to four different odors, we performed PCA. The distributions of the first two PCs (PC 1, **G**; PC 2, **H**) are displayed as box plots. The PC 1 and PC 2 explain 22.92 and 6.75% of the variability of responses, respectively. In **(E–H)**, the line in the box and the box represents the median and the quartiles, respectively. Outliers are shown as dots.

We summarized the temporal activity patterns of type1 and type2 PNs recruited by a given odor stimulus (**Figure 5**). The responses to hexanol (28 responses from 14 type2 PNs; 40 responses from 20 type1 PNs) and to cineol (38 responses from 19 type2 PNs; 46 response from 23 type1 PNs) are shown as raster plots and accumulated histograms in **Figures 5A–D**. We statistically compared histograms during the period of odor stimulation (gray bar in **Figures 5A–D**) and revealed that a given odor elicited both type1 and type2 PNs with significantly different temporal patterns (KS-test: hexanol, *P* = 1.01 × 10^-11^, *D* = 0.51; cineol, *P* = 1.97 × 10^-10^, *D* = 0.48). In both odor stimuli, differences of temporal activity patterns between type1 and type2 PNs reached peaks at two different timings: a period of 100–130 ms and a period of 250–290 ms after the odor onset. The former and the later peaks appeared to reflect the differences in response latencies and durations between type1 and type2 PNs.

To compare odor-induced temporal activity patterns across multiple PN responses, we performed cluster analyses and PCAs using PSTHs with a bin of 20 ms (**Figures 5E,F** and **Supplementary Figures S2, S3**). Based on cluster dendrograms, PSTHs of 68 PN responses to hexanol and 84 PN responses to cineol were, respectively, classified into four clusters (**Supplementary Figures S2, S3**). In both odor stimuli, responses of type2 PNs were grouped together into one or two clusters (hexanol, cluster 3; cineol, clusters 3 and 4), whereas those of type1 PNs were distributed throughout four clusters (**Figures 5E,F** and **Supplementary Figures S2, S3**). Consistent with cluster analysis results, the first two PCs (PC 1 and PC 2) were narrowly distributed in type2 PNs and broadly distributed in type1 PNs (**Figures 5E,F**). The PC variances were significantly different between type1 and type2 PNs (*F*-test; hexanol, PC 1, *P* = 2.15 × 10^-6^, *F* = 6.65, PC 2, *P* = 2.15 × 10^-6^, *F* = 3.16; cineol, PC 1, *P* = 0.0060, *F* = 2.50, PC 2, *P* = 0.00011, *F* = 3.74). These results strongly suggested that a given odor elicited similar temporal response patterns in different type2 PNs, whereas a given odor-induced temporally diverse activity patterns in different type1 PNs.

**FIGURE 6:**
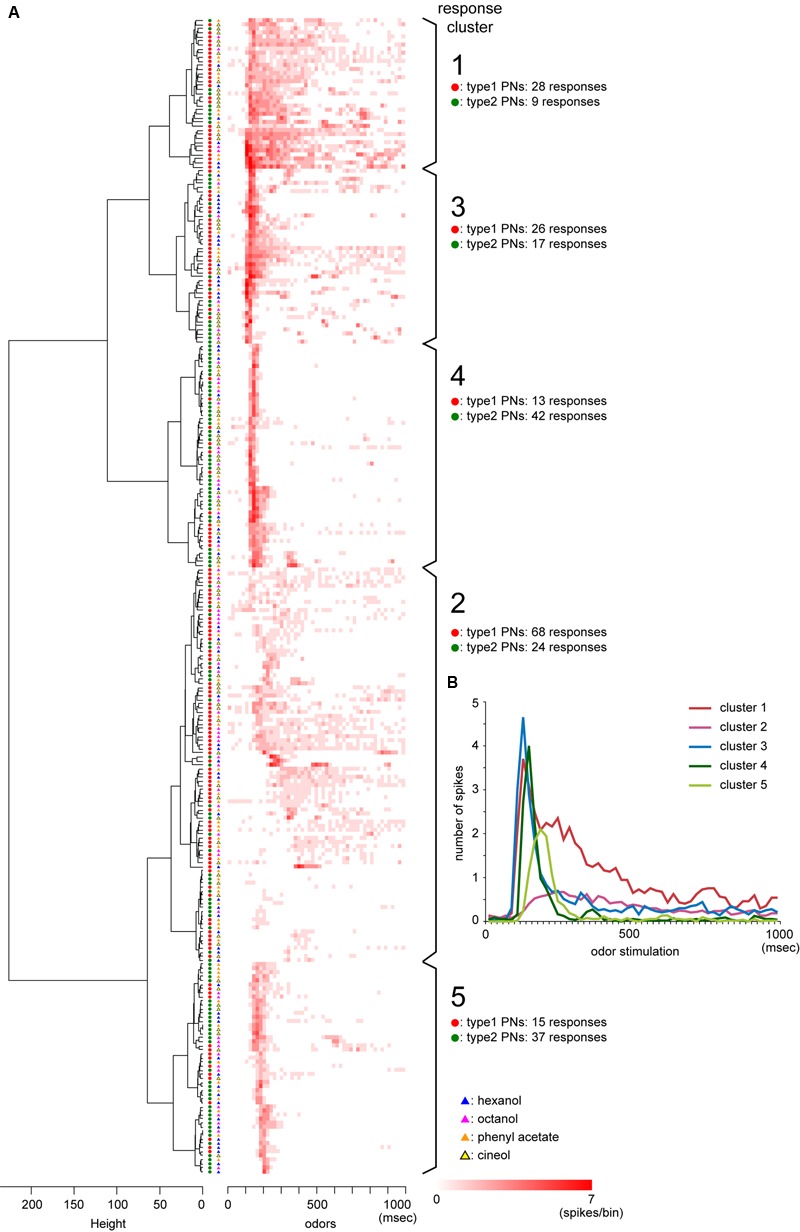
**Clustering of temporal activity patterns of PNs. (A)** Results of cluster analysis. We classified temporal activity patterns of 284 PN responses into five response clusters based on the cluster dendrogram (left panel) formed by Ward’s method. The heat map shows PSTHs with a bin of 20 ms, and the heater color represents the higher spike activities within the bin. Red and green circles represent responses from type1 and type2 PNs, respectively. Responses to hexanol, octanol, phenyl acetate, and cineol are, respectively, denoted as blue, pink, orange, and yellow triangles. **(B)** Averages of PSTHs in each of five response clusters.

To evaluate temporal activity patterns induced by different odors, we classified 284 PN responses induced by four different odors (hexanol, octanol phenyl acetate, and cineol) into five clusters (**Figure 6**). Among the five clusters, clusters 1 and 2, which showed phasic-tonic spike activities, were predominantly assigned to type1 PN responses, whereas clusters 4 and 5, which showed on-phasic spike activities, were predominantly assigned to type2 PN responses (**Figure 6**). Thus, type1 and type2 PN responses were clustered into different groups (χ^2^-test, *P* = 2.05 × 10^-10^, *df* = 4, χ^2^= 51.18). Conversely, five response clusters were not affected by differences in odor stimuli (χ^2^-test, *P* = 0.11, *df* = 12, χ^2^= 18.23). PCA revealed that the first two PCs (PC 1 and PC 2) were narrowly distributed in type2 PNs and broadly distributed in type1 PNs (**Figures 5G,H**). The PC 1 variances were significantly different across PN types, but not across odors (two-way ANOVA; odors, *P* = 0.319, *df* = 3, *F* = 1.18; PN types, *P* = 4.74 × 10^-11^, *df* = 1, *F* = 46.95). These results strongly suggested that different odors induced similar temporal activity patterns in type2 PNs and varied temporal activity patterns in type1 PNs.

### Odor-Specific Early and Late Responses of Type2 PNs

**FIGURE 7:**
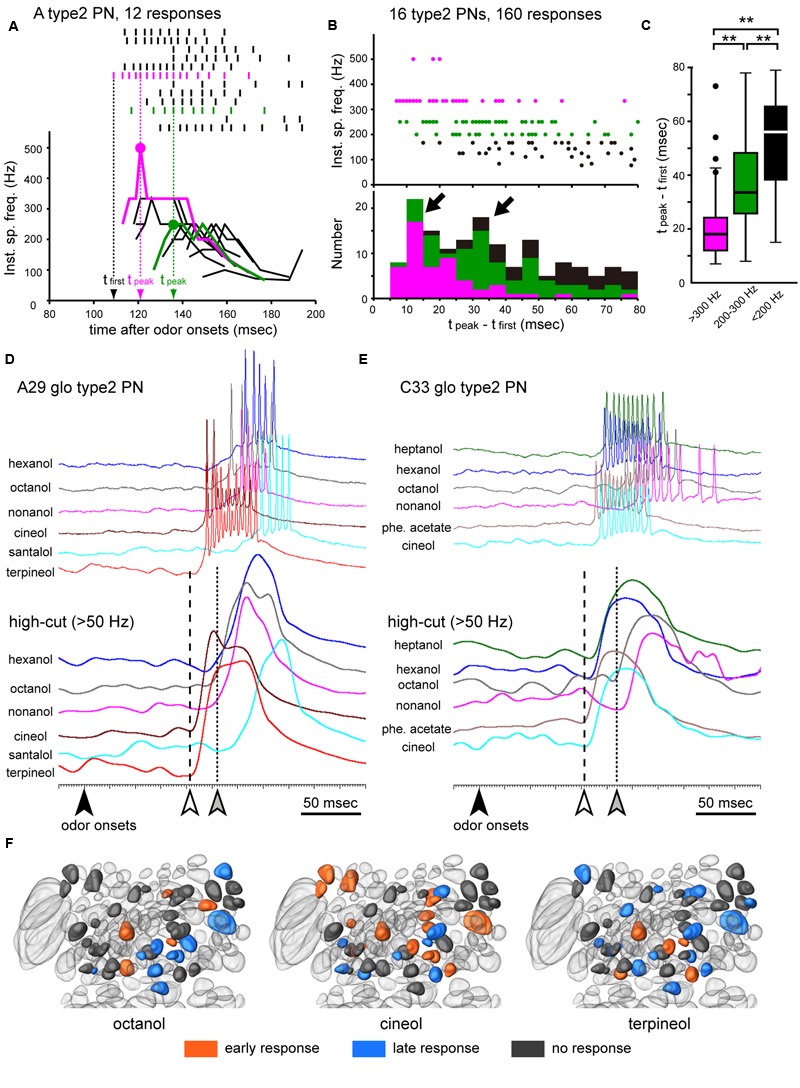
**Early and late responses in type2 PNs. (A)** Temporal dynamics in odor-induced action potentials of a type2 PN. On-phasic responses to six different odors (12 responses) are shown as raster plots (top) and time-courses of instantaneous spike frequencies (bottom). In each response, we identified a “peak spike” (dots; see “Materials and Methods”). Magenta and green responses indicate typical early and late responses, respectively. **(B,C)** Time distribution of peak spikes obtained from 160 responses. We identified peak spikes from 160 olfactory responses recorded from 16 different type2 PNs. The timing of each peak spike is corrected as follows; t_peak_ – t_first_, where t_peak_ is a time of the peak spike from odor onset and t_first_ is a time of earliest odor-induced spike in the specimen (exampled in **A**). A total of 160 peak spikes are plotted in a scatter diagram (upper in **B**), and the instantaneous spike frequencies ranged from >300 Hz, 200–300 Hz, and <200 Hz, which are colored as magenta, green, and black dots, respectively. In the histogram (bottom in **B**), the number of peak spikes are counted every 5 ms after t_first_, and there are two prominent peaks of the histogram (arrows in **B**). Peak spikes with high instantaneous spike frequencies (>300Hz; magenta boxes in **B,C**) are distributed significantly earlier than those with low instantaneous spike frequencies (200–300 Hz; green boxes in **B,C**). In **(C)**, the line in the box and the box represents the median and the quartiles, respectively. Outliers are shown as dots. Results of statistical comparison using ANOVA and *post hoc* Tukey test are shown as asterisks (*t*-test, ^∗∗^*P* < 0.01). **(D,E)** Olfactory responses of two different type2 PNs. To show rising points of early (white arrowheads) and late responses (gray arrowheads), excitatory responses elicited by different odor stimuli (top) are processed by a low-pass filter set at 50 Hz (bottom). Olfactory responses are arrayed based on odor onsets (black arrowheads). **(F)** Response type-specific glomerular organization. Glomeruli innervated by recorded type2 PNs are colored according to response types. When type2 PNs exhibited early or late responses to a given odor (octanol, cineol, or terpineol), glomeruli innervated by the PNs are colored orange or blue, respectively. Glomeruli innervated by PNs that did not show any excitatory responses to the odor are colored gray.

Type2 PNs generally exhibited phasic on-responses with short latencies. However, cluster analysis of PSTHs suggested that phasic on-responses of type2 PNs were further classified into two different temporal patterns: clusters 4 and 5 in **Figure 6A**. Averages of PSTHs in each cluster revealed that responses in cluster 4 exhibited stronger spike activities with earlier latencies than those in cluster 5 (**Figure 6B**). To describe temporal activity patterns of type2 PNs in more detail, we analyzed odor-induced spike arrays in 160 phasic on-responses obtained from 16 different type2 PNs that exhibited excitatory responses to more than five different odors (**Figures 7A–C**). In each response, we identified a spike at which instantaneous spike frequencies peaked or plateaued, which was termed a “peak spike” (dots in **Figure 7A**). Time distribution of 160 peak spikes revealed that the phasic on-responses of type2 PNs were classified into two types: early and late responses (arrows in **Figure 7B**), exactly corresponding to the clusters 4 and 5 (**Figure 6A**). Additionally, peak spikes with high spike frequencies (>300 Hz; magenta in **Figures 7B,C**) were distributed significantly earlier than those with low spike frequencies (200-300 Hz; green in **Figures 7B,C**; ANOVA and *post hoc* Tukey test, *P* = 4.0 × 10^-7^, *df* = 16.25). These results also suggested that type2 PNs exhibited early and strong response or late and weak response to a given effective odor.

Early and late responses were commonly observed in type2 PNs which exhibited excitatory responses to multiple odors, and the latencies of late responses were 30–40 ms longer than the early responses (**Figures 7D,E**). In each specimen, the EPSP rise was almost identical for both early and late responses elicited by different odors (lower traces in **Figures 7D,E**), suggesting that specific neural mechanisms underlie early and late responses in type2 PNs. In individual type2 PNs, an average of 1.9 odors induced late responses, and an average of 1.1 odors induced early responses. Thus, odor-specificity of the early response was significantly greater than that of the late response (Wilcoxon-test, *N* = 63, *P* = 2.8 × 10^-4^). In the case of a type2 PN innervating the A29 glomerulus (**Figure 7D**), two odors (cineol and terpineol) elicited early responses (white arrowhead in **Figure 7D**), and four odors (hexanol, octanol, nonanol, and santalol) induced late responses (gray arrowhead in **Figure 7D**). In contrast, a type2 PN with dendrites in the C33 glomerulus exhibited early responses to heptanol, hexanol, phenyl acetate, and cineol (white arrowhead in **Figure 7E**), and exhibited late responses to octanol and nonanol (gray arrowhead in **Figure 7E**). Thus, the response type evoked by a given odorant differed depending on the innervating glomerulus (**Figure 7F**). Therefore, our results suggested that the odor-induced activity pattern of a population of type2 PNs drastically changed within a brief time window (**Figure 7F**).

### Simultaneous Intracellular Recordings from Two Different PNs

Intracellular recordings revealed that a given odor stimulus activated both type1 and type2 PNs with different temporal patterns (**Figures 4–7**). In addition, there were early and late on-phasic responses in type2 PNs (**Figures 6, 7**). To investigate the neural mechanisms underlying temporal activity patterns of PNs, we performed simultaneous intracellular recordings from a pair of type1 and type2 PNs.

**FIGURE 8:**
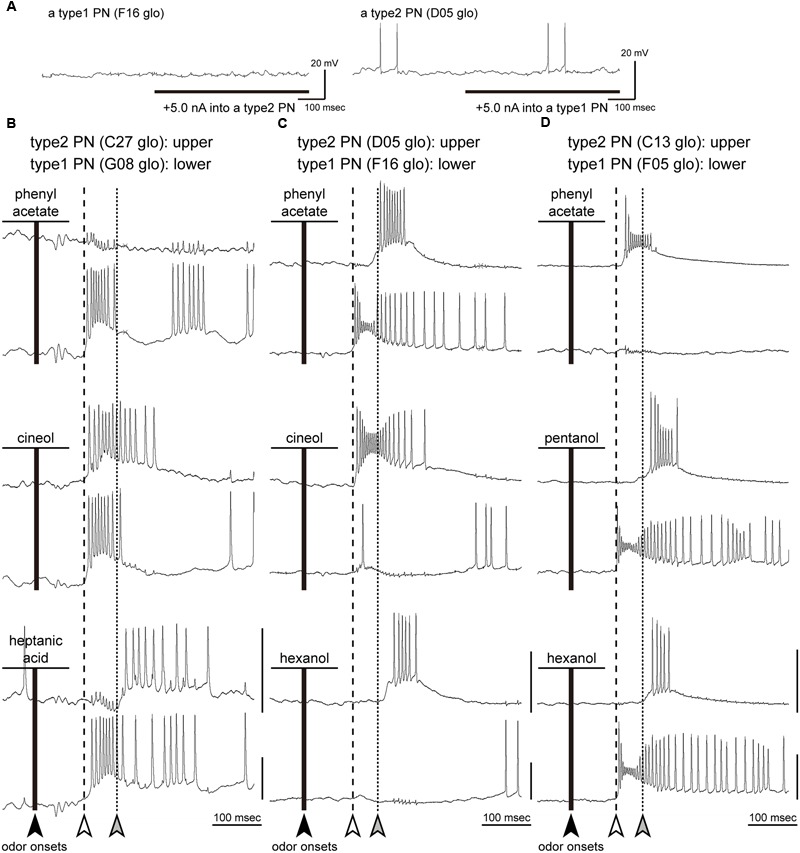
**Simultaneous intracellular recordings from a type1 PN and a type2 PN. (A)** Current injection to a PN. The artificial excitation of one PN by an inward current injection (5.0 nA) did not affect the membrane potential of the other PN. **(B–D)** Three pairs of response traces of simultaneous recordings from a type2 PN (upper traces) and a type1 PN (lower traces). The glomeruli innervated by PNs are denoted in each panel. We selected typical combinational responses elicited by three different odorants (black lines). Olfactory responses are arrayed based on timings of odor onsets (black arrowheads). Based on type2 PN response patterns, we identified latencies of early (white arrowheads and broken lines) and late responses (gray arrowheads and dotted lines) in each simultaneous recording. In each trace, there was considerable cross-talk (1–5 mV) between the two simultaneously recorded signals. Vertical bars = 20 mV.

We successfully conducted simultaneous intracellular recordings from six pairs of type1 and type2 PNs. In each recording, some odors elicited different excitatory responses in both type1 and type2 PNs (**Figure 8**). We recorded 39 combinational excitatory responses from six pairs of type1 and type2 PNs. Among them, **Figure 8** shows the combinational response patterns induced by three different odors obtained from three simultaneous intracellular recordings. In each of the traces, considerable cross-talk (1–5 mV) between two simultaneously recorded signals was identified. In these recordings, the artificial excitation of one PN by an inward current injection (5.0 nA) did not affect the membrane potential of the other PN (**Figure 8A**). This suggested that there were no direct synaptic connections between the two PNs, consistent with results from post-recording visualizations (data not shown).

**FIGURE 9:**
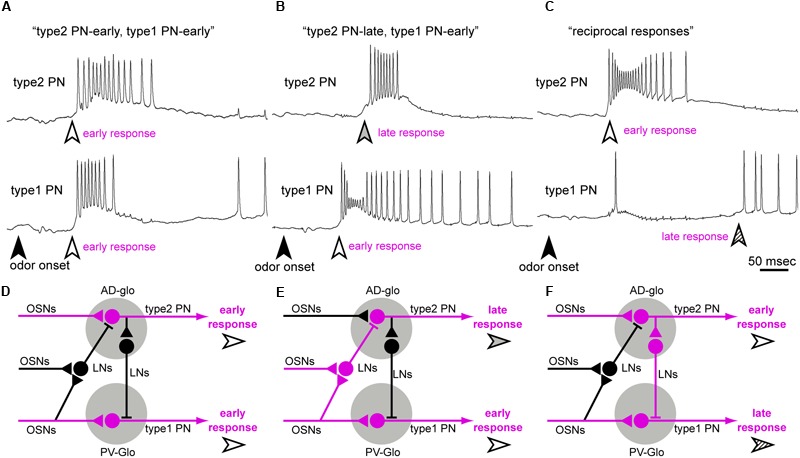
**Putative neural models to elicit olfactory on-responses of type2 and type1 PNs. (A–C)** Three typical combinations of temporal activity patterns revealed by simultaneous recordings from a type2 PN (top) and a type1 PN (bottom). When a given odor stimulus activated both type1 and type2 PNs, combinational response patterns are categorized into one of three typical patterns: “type2 PN-early and type1 PN-early responses” **(A)**, “type2 PN-late and type1 PN-early responses” **(B)** and “reciprocal responses” **(C)**. White and gray arrowheads, respectively, indicate latencies of early and late responses of type2 PNs. Type1 PNs often fired after a break phase (hatched arrowhead in **C**). **(D–F)** Putative models to elicit three typical combinational response patterns of type2 and type1 PNs. A given odorant activated specific neurons (magenta lines) and activated type2 and type1 PNs with different temporal patterns. Based on results of transmission electron microscopy (Distler and Boeckh, 1997a,b) and electrophysiologies from OSNs (Fujimura et al., 1991) and LNs (Husch et al., 2008, 2009; Watanabe et al., 2012a), we assumed excitatory (triangles) and inhibitory (t-shaped bar) synapses in the AL. AD-glo, anterior-dorsal group glomerulus; PV-glo, posterior-ventral group glomerulus.

Based on the response patterns of a type2 PN, we identified latencies of early responses (white arrowheads and broken lines in **Figure 8**) and late responses (gray arrowheads and dotted lines in **Figure 8**) in each simultaneous recording. When a given odor stimulus elicited excitatory responses in both type1 and type2 PNs, the combinational response patterns were categorized into one of three typical patterns (**Figures 9A–C**). First, both type 1 and type2 PNs started to fire nearly simultaneously at the latency of early responses (responses to cineol shown in **Figure 8B**). Among the 39 combinational excitatory responses, 11 responses from 4 PN pairs were categorized into the pattern. There were no events prior to the early responses in both PN types. Second, the type1 PN fired with an early response, then the type2 PN fired with a late response (responses to heptanoic acid in **Figure 8B**, to phenyl acetate in **Figure 8C**, and to pentanol and hexanol in **Figure 8D**). Among the 39 combinational excitatory responses, 18 responses exhibited the pattern. This combinational response patterns were commonly observed in all PN pairs. Interestingly, the late responses were observed only in type2 PNs. Third, type1 and type2 PNs often exhibited excitatory responses reciprocally (responses to cineol and hexanol in **Figure 8C**). The type1 PN did not exhibit spike activities during the firing of the type2 PN, and then fired with a low spike frequency after finishing the type2 PN response. Among the 39 combinational excitatory responses, 10 responses from 4 PN pairs were categorized into the pattern. Based on the present results and previous anatomical and physiological reports in the cockroach, we propose that neural circuits in the cockroach AL are involved in odor-induced temporal activity patterns in both types of PNs (See “Discussion,” **Figure 9**).

## Discussion

Cockroaches exhibit excellent olfactory discrimination and learning capabilities (Sakura et al., 2002; Watanabe et al., 2003). Higher brain centers receive odor information from a population of PNs to discriminate odors, and to acquire and retrieve olfactory memories (Watanabe et al., 2011). In the cockroach, general odors are processed in two major types of uniglomerular PNs, type1 and type2 PNs, which have been anatomically identified from distinct termination areas in the MB calyces (Strausfeld and Li, 1999a). The current results revealed that axon terminals of type1 and type2 PNs are spatially segregated in the MB calyces, as well as in the LH. In addition, type1 and type2 PNs arborize in the postero-ventral and antero-dorsal group glomeruli, respectively. The former glomeruli specifically receive olfactory inputs from antennal perforated basiconic sensilla and the latter glomeruli from trichoid and grooved basiconic sensilla (Watanabe et al., 2012b). These results indicate that the olfactory system of a phylogenetically basal insect, the cockroach, is anatomically segregated into two distinct parallel pathways from the peripheral sensory system to higher brain centers. Additionally, comprehensive intracellular recording revealed that type1 PNs and type2 PNs exhibited different odor-specificities in response to the nine tested odorants. In particular, quantitative analyses and simultaneous intracellular recording clearly reveled that a given odor-activated PNs in both pathways with different temporal patterns. Anatomical and physiological differences between type2 and type1 PNs are summarized in **Table 1**. These results strongly suggest that two parallel pathways have different odor coding strategies.

**Table 1:** Anatomical and physiological differences between type1 and type2 projection neurons (PNs).

	Type 2 PNs		Type 1 PNs	
*Input*				
Glomerular groups	Antero-dorsal group glomeruli		Postero-ventral group glomeruli	
	T1–T4 groups		T5–T7 groups	T8–T10 groups
Antennal sensilla	Perforated basiconic sensilla		Trichoid sensilla	Grooved basiconic sensilla
*Output*				
Mushroom bodie (MB) calyces	zone I		zones III and IIIA	
Lateral horn (LH)	Antero-dorso-lateral region		Central region	
*Response properties*				
Odor-specificity	low		low	high
Temporal pattern	On-phasic		Various	
	Early response	Late response		

The parallel pathways for processing general odors have been studied in hymenopteran insects, which are equipped with m-ALT and l-ALT PNs (Kirschner et al., 2006; Zube et al., 2008; Zwaka et al., 2016). In honey bees, a given odorant activates both l-ALT and m-ALT PNs, and these neurons exhibit different physiological properties according to odor-specificities and concentrations (Muller et al., 2002; Krofczik et al., 2008; Yamagata et al., 2009; Brill et al., 2013; Carcaud et al., 2015). The innervating glomeruli and projection patterns suggest that m-ALT and l-ALT PNs in hymenopteran insects might correspond to type1 and type2 PNs in the cockroach, respectively. However, there are substantial anatomical differences between these insects. First, cockroaches lack uniglomerular PNs running through the l-ALT. Second, OSNs in single pore-plate sensilla send axons to both PN types in honey bees (Kelber et al., 2006; Kropf et al., 2014). Third, terminal regions of the two PN types partially overlap in honey bees. To date, two parallel pathways to process general odors have been reported only in the cockroach (the current study) and hymenopteran insects (Galizia and Rössler, 2010; Martin et al., 2011). These phylogenetically distinct insect groups may have independently evolved parallel olfactory pathways, and may be an example of convergent evolution. Future studies examining olfactory pathways in other insects are needed to confirm this hypothesis.

### Response Properties of Each PN Type in the Cockroach Brain

We showed that odor-specificities of type1 PNs to nine tested odorants were higher than in type2 PNs. We selected nine odorants, each eliciting strong excitatory effects in one of the eight OSN groups (Fujimura et al., 1991). Among these odorants, seven were putative ligands of perforated basiconic sensilla. Therefore, it is not surprising to find that type2 PNs, which receive sensory inputs from OSNs in the basiconic sensilla, were broadly tuned to tested odorants. Conversely, santalol and heptanoic acid are selective ligands of the grooved basiconic sensilla, and OSNs in the sensilla tend to terminate in T8–T9 group glomeruli, in which narrowly tuned type1 PNs arborize (Watanabe et al., 2012b). Thus, response ranges of PNs are strongly affected by sensory inputs from OSNs. However, specific ligands of perforated basiconic sensilla, such as low molecular alcohols, activate not only type2 PNs but also type1 PNs and *vice versa*. This suggests that PNs might be more broadly tuned to odors than their presynaptic OSNs, as reported in *Drosophila* AL, indicating that the cockroach and *Drosophila* AL may share a similar form of lateral excitation through the recurrent pathways via local neurons (Wilson et al., 2004).

Interestingly, results showed that type1 PNs arborizing in T5–T7 group glomeruli are broadly tuned to nine tested odors, including PNs that responded to all tested odors. In the cockroach, the T5–T7 group glomeruli receive sensory inputs from trichoid sensilla (Watanabe et al., 2012b), and a part of trichoid sensilla contain OSNs that respond to the onset and offset of odor stimulus (Hinterwirth et al., 2004; Burgstaller and Tichy, 2011). This suggests that type1 PNs process more specific aspects of odor information compared with type2 PNs. Among the nine tested odors, the heptanoic acid selectively activated type1 PNs (**Figure 3B**). In the cockroach, behavioral repercussions of the heptanoic acid remain unclear, but some types of fatty acids, such as butyric acid, hexadecanoic acid, and pentadecanoic acid, exhibit attractive effects (Imen et al., 2015). In addition, type1 PNs with dendrites in T10 glomeruli exhibited excitatory responses to temperature and humidity changes (Nishino et al., 2003). M-ALT PNs in hymenopteran insects, which are analogs of type1 PNs in the cockroach, include PNs that process specific odors, such as cuticular hydrocarbons in ants (Nishikawa et al., 2012) and blood pheromones in honey bees (Carcaud et al., 2015). It was recently shown that m-ALT PNs are critical for successful appetitive olfactory learning (Carcaud et al., 2016).

In each type2 PN, effective odor stimuli elicited on-phasic responses with different latencies: early and late responses. In each recording, the temporal structures of early (or late) responses elicited by different odorants were almost identical. This finding suggests that individual type2 PNs are difficult to encode odor qualities in the temporal response patterns. Conversely, cluster analysis revealed latencies and temporal activity patterns of early and late responses remained consistent among different type2 PNs. This pattern suggests that a given odor stimulus might activate many different type2 PNs with short or long latencies, and odor quality might be encoded as the extent of synchronous activity of populations of type2 PNs. Because the MB intrinsic neurons (KCs) have been reported to detect synchronized inputs from PNs in the cockroach (Demmer and Kloppenburg, 2009), inputs from type 2 PNs may be more suitable for exciting KCs compared to those from type 1 PNs.

In contrast, type1 PNs exhibited long-lasting responses during the odor stimulus period, and different odor stimuli induced different response latencies and different temporal activity patterns in each type1 PN. Thus, individual type1 PNs may encode odor-specificities in their temporal activity patterns. In fact, odor-specificity in type1 PNs, especially type1 PNs that innervate the T8–T10 glomerular groups, are higher than that in type2 PNs. In this study, we performed single-cell based analyses to reveal similarities and diversities of temporal activity patterns across PNs. However, the functional meaning of the temporal activity patterns remains unknown. PN population-based analyses are needed in the future to reveal the differences in parallel coding strategies in the cockroach brain.

### Putative Neural Models of Early Olfactory Processing in the Cockroach AL

In simultaneous recordings, we identified three typical combinational odor-induced activity patterns between a type1 PN and a type2 PN: “type2 PN-early and type1 PN-early responses,” “type2 PN-late and type1 PN-early responses,” and “reciprocal responses” (**Figures 9A–C**). Previous extracellular recordings revealed that odors used in this study elicit phasic-tonic spike activities in OSNs in the cockroach; each OSN exhibits a strong phasic on-response and a prolonged weak tonic response that outlasts the period of effective odor stimulation (Fujimura et al., 1991; Prof. Yokohari, personal communications). Based on these results and previous anatomical and physiological studies in the cockroach (Ernst and Boeckh, 1983; Distler and Boeckh, 1997a,b; Husch et al., 2008, 2009; Watanabe et al., 2012a), we propose a neural model of the mechanisms underlying on-responses of both PN types (**Figures 9D–F**). In the “type2 PN-early and type1 PN-early responses,” early responses of both PNs arise at the same latencies to a given odor, with no neural events before the early responses. Thus, we hypothesize that early responses in both PN types are elicited by direct inputs from activated OSNs (**Figure 9D**).

In simultaneous recording, we often observed reciprocal interactions between type1 and type2 PNs that may be mediated by neural circuits interconnecting the two pathways in the AL. The “type2 PN-late and type1 PN-early responses” were observed in all pairs of type1 and type2 PNs (**Figure 8**). We occasionally observed putative hyperpolarizing membrane potential before the onset of the late response of the type2 PNs. This strongly suggests that inhibitory neural circuits in the cockroach AL might mediate late responses of type2 PNs. Interestingly, the late responses were observed only in type2 PNs. Therefore, we hypothesize that the feed-forward inhibitory pathways, which receive olfactory inputs from OSNs in the postero-ventral group glomeruli and terminate at the antero-dorsal group glomeruli (**Figure 9E**). Previous cockroach studies reported that more than 90% of LNs exhibit multiglomerular projections, with dendritic arborizations in most, but not all glomeruli, termed LN1s (Distler and Boeckh, 1997b; Husch et al., 2008, 2009; Watanabe et al., 2012a). Almost all LN1s are GABAergic, and make synapses with both OSNs and PNs (Distler and Boeckh, 1997b). In a previous study, simultaneous recordings of two different LN1s revealed that LN1s exhibit excitatory on-responses to effective odors, and odor-induced spikes are temporally synchronized between two LN1s (Watanabe et al., 2012a). Since more than 25 GABAergic LN1s are assumed to converge onto a glomerulus in the cockroach (Distler and Boeckh, 1997b), synchronized firing of multiple LN1s induced strong inhibitory effects on postsynaptic neurons. Based on the previous findings, we hypothesize that late responses of type2 PNs are mediated by synchronized firing of multiple GABAergic LN1s in the cockroach AL.

In addition, in response to a given odor, a type1 PN often exhibit hyperpolarization during activation of a type2 PN, a phenomenon we refer to as “reciprocal responses” (**Figure 9F**). This suggests that type1 PNs might receive inhibitory inputs from activated type2 PNs. Because there are no direct connections between type1 and type2 PNs in the cockroach AL, “reciprocal responses” might be mediated by LNs. After the break phase, type1 PNs fired with low spike frequencies, and they might be induced by the post inhibitory rebound excitation and/or prolonged inputs from activated OSNs. In the cockroach AL, several LN types, except for LN1s, have been physiologically and anatomically identified (Husch et al., 2009; Fusca et al., 2015). To better understand the olfactory processing pathways in the cockroach AL, simultaneous intracellular recordings should be performed from a PN and a LN.

The current findings raise the question of what odor stimulus parameters are extracted by PNs. It remains unknown, although two different response phases of type2 PNs may provide valuable insights. If our hypothesis is true, the early responses might be driven by direct excitatory inputs from OSNs, whereas the late responses are mediated by feed-forward pathways. Thus, the latency and strength of early responses appear to reflect the timing and concentration of odor stimuli coded by OSNs, respectively. In contrast, feed-forward pathways through multiglomerular LNs integrate sensory inputs from many OSNs, suggesting that late responses of type2 PNs might be better suited for processing of odor mixture. In fact, in each type2 PN, odor-specificity of the late response was lower than in the early response.

It remains to be determined how do cockroaches process olfactory information from type1 and type2 PNs in the higher brain centers. In the cockroach, KCs require convergent and synchronous inputs from multiple PNs for firing (Demmer and Kloppenburg, 2009). Additionally, inputs from PNs to KCs are modulated by four GABAergic calycal giant neurons (CGs) with dendrites in the termination fields of MB output neurons (Nishino et al., 2012b; Takahashi et al., 2017). Recent anatomical evidence suggests that olfactory inputs from type1 and type2 PNs to the MB calyces are modulated by different subset of CGs (Takahashi et al., 2017). Specifically, the axon terminal of three CGs, including a non-spiking neuron, are found to converge on the terminal region of type2 PNs, forming fine and complex negative-feedback circuits. It has been reported in the locust *Schistocerca americana* that a non-spiking GABAergic feedback neuron provides powerful and phase-locked inhibition to KCs in MB calyces, reinforcing the ability of KCs to detect coincident spikes from PN populations (Papadopoulou et al., 2011). In addition, a CG that may modulate type1 PNs is much larger than the remaining three CGs, suggesting that it receives predominately more MB output neurons than other CGs. These results suggest that inputs from type1 PNs to KCs may be influenced by MB activities, such as olfactory learning and memory. Overall, the current results suggest that the cockroach uses two parallel coding strategies for processing general odor.

## Author Contributions

All authors had full access to all the data in the study and take responsibility for the integrity of the data and the accuracy of the data analysis. Study concept and design: HW. Acquisition, analysis and interpretation of data: HW. Drafting of the article: HW. Critical revision of the article for intellectual content: HW, HN, MM, and FY. Obtained funding: HW, HN, MM, and FY. Administrative, technical, and material support: HW and HN. Study supervision: MM and FY.

## Conflict of Interest Statement

The authors declare that the research was conducted in the absence of any commercial or financial relationships that could be construed as a potential conflict of interest.

## Supplementary Material

FIGURE S1 **Cluster analysis of recorded projection neurons (PNs).** We performed the cluster analysis (Ward’s method) using response intensities to nine odorants. We classified 178 PNs into five odor spectra groups. The five odor spectra groups are arbitrarily grouped based on the dendrogram (left panel) and response intensities (heat map). Response intensities to nine odors were summarized as heat colors.

FIGURE S2 **Cluster analysis of temporal activity patterns elicited by hexanol.** Based on peri-stimulus time histograms (PSTHs) during the 1-s hexanol stimulations, we classified 68 PN responses into four response clusters. The four response clusters are arbitrarily grouped based on the cluster dendrogram (left panel, height 30) formed by Ward’s method and PSTHs. The heat map shows PSTHs with a bin of 20 ms, and the heater color represents higher spike activities within the bin. Red and green circles represent the responses from type1 and type2 PNs, respectively.

FIGURE S3 **Cluster analysis of temporal activity patterns elicited by cineol.** Based on PSTHs during the 1-s cineol stimulations, we classified 84 PN responses into four response clusters. The four response clusters are arbitrarily grouped based on the cluster dendrogram (left panel, height 30) formed by Ward’s method and PSTHs. The heat map shows PSTHs with a bin of 20 ms, and the heater color represents the higher spike activities within the bin. Red and green circles represent the responses from type1 and type2 PNs, respectively.
