# Supplementary figures and images for: Two Parallel Olfactory Pathways for Processing General Odors in a Cockroach
Source: Front Neural Circuits. 2017 May 5;11:32. doi: 10.3389/fncir.2017.00032 (PMC5418552; doi:10.3389/fncir.2017.00032)

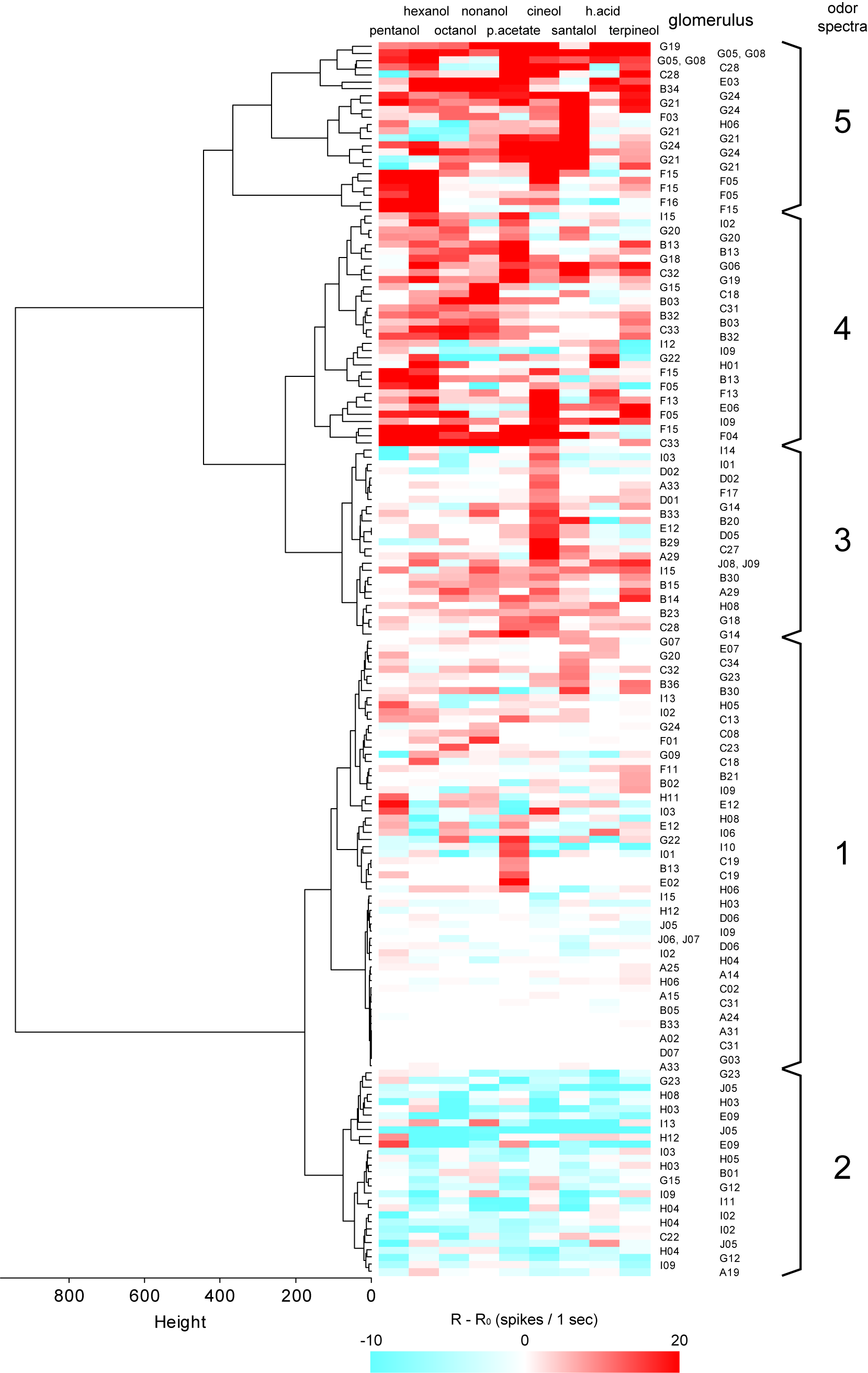

Supplement: FIGURE S1 — Cluster analysis of recorded projection neurons (PNs). We performed the cluster analysis (Ward’s method) using response intensities to nine odorants. We classified 178 PNs into five odor spectra groups. The five odor spectra groups are arbitrarily grouped based on the dendrogram (left panel) and response intensities (heat map). Response intensities to nine odors were summarized as heat colors. [file Image_1.TIF]

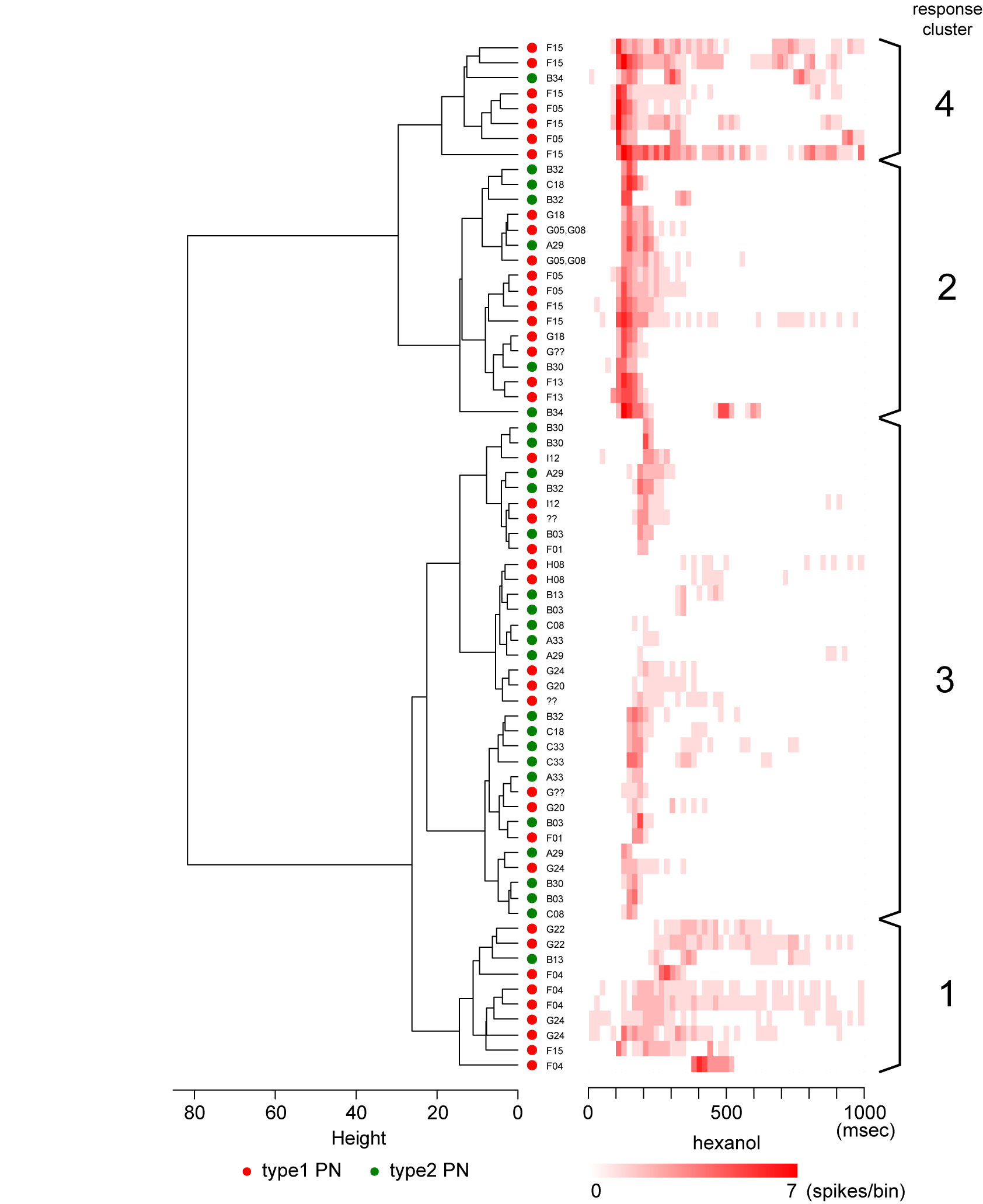

Supplement: FIGURE S2 — Cluster analysis of temporal activity patterns elicited by hexanol. Based on peri-stimulus time histograms (PSTHs) during the 1-s hexanol stimulations, we classified 68 PN responses into four response clusters. The four response clusters are arbitrarily grouped based on the cluster dendrogram (left panel, height 30) formed by Ward’s method and PSTHs. The heat map shows PSTHs with a bin of 20 ms, and the heater color represents higher spike activities within the bin. Red and green circles represent the responses from type1 and type2 PNs, respectively. [file Image_2.TIF]

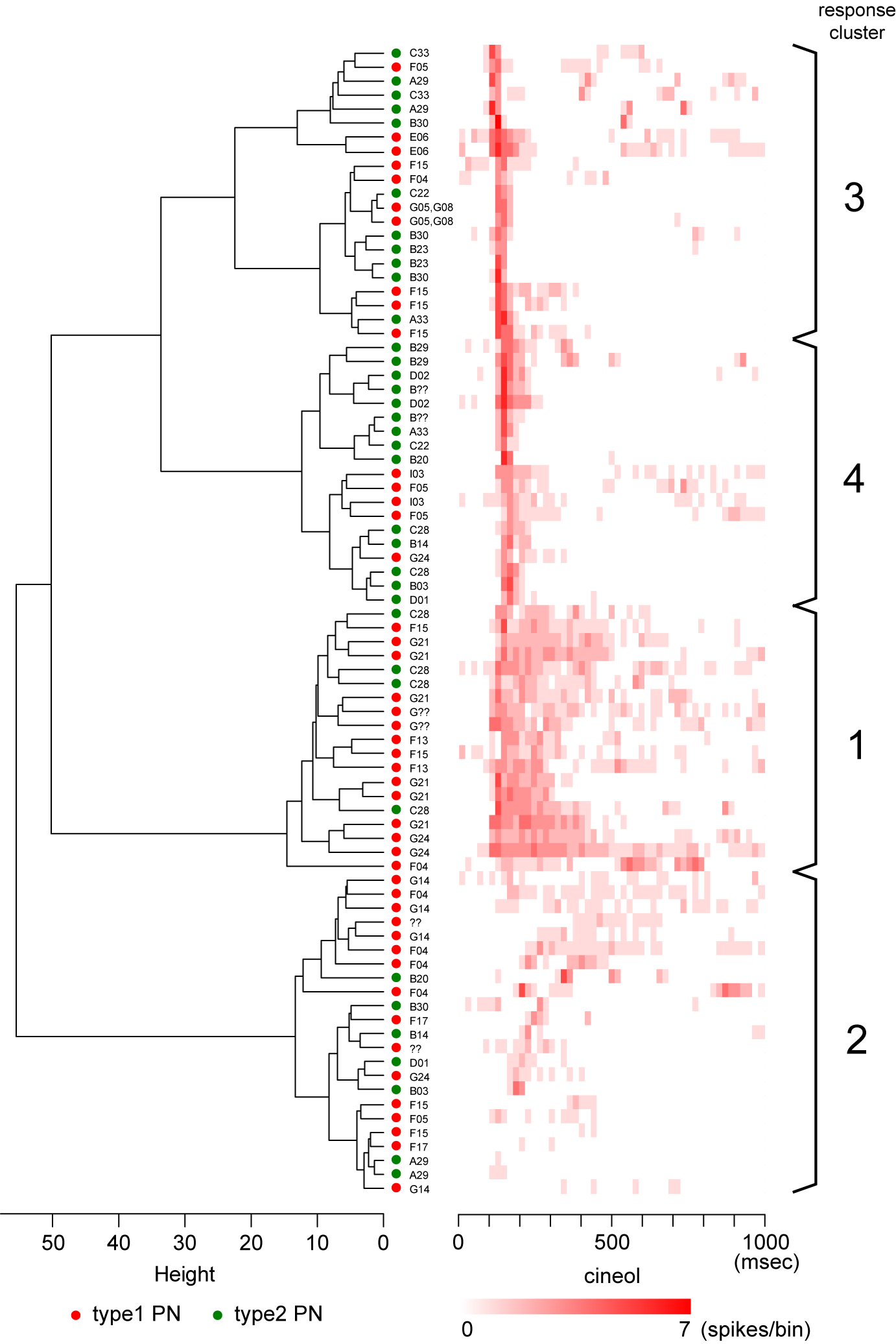

Supplement: FIGURE S3 — Cluster analysis of temporal activity patterns elicited by cineol. Based on PSTHs during the 1-s cineol stimulations, we classified 84 PN responses into four response clusters. The four response clusters are arbitrarily grouped based on the cluster dendrogram (left panel, height 30) formed by Ward’s method and PSTHs. The heat map shows PSTHs with a bin of 20 ms, and the heater color represents the higher spike activities within the bin. Red and green circles represent the responses from type1 and type2 PNs, respectively. [file Image_3.TIF]
